# Effects of Movement Retraining and Lumbar Stabilization Exercises in Mechanical Low Back Pain: A Pilot Study

**DOI:** 10.7759/cureus.54291

**Published:** 2024-02-16

**Authors:** Roopa Desai, Manisha Rathi, Tushar J Palekar

**Affiliations:** 1 Musculoskeletal Sciences, Dr. D. Y. Patil College of Physiotherapy, Dr. D. Y. Patil Vidyapeeth, Pune, IND; 2 Physiotherapy, Dr. D. Y. Patil College of Physiotherapy, Dr. D. Y. Patil Vidyapeeth, Pune, IND; 3 Physical Medicine and Rehabilitation, Dr. D. Y. Patil College of Physiotherapy, Dr. D. Y. Patil Vidyapeeth, Pune, IND

**Keywords:** movement retraining, uncontrolled movements, core strength, low back pain, lumbar stabilization exercises, movement control

## Abstract

Objective

To determine and compare the effects of movement retraining (MR), lumbar stabilization exercises (LSE), and a combination of both these exercises on pain, flexibility, strength, and functional disability in chronic mechanical low back pain (CMLBP) patients.

Materials and methods

Fifteen CMLBP participants, aged 20-40 years, were randomly allocated into three groups. Group A (n=5) received MR, group B (n=5), LSE, and group C (n=5), a combination of MR and CSE, along with hot packs for eight weeks, thrice a week on alternate days. Outcomes used were the Numerical Pain Rating Scale (NPRS), Modified Modified Schober’s Test (MMST), Pressure Biofeedback (PBU), Roland Morris Disability Questionnaire (RMDQ), and Movement Control (MC) dissociation tests to identify MC impairments and were assessed at pre-intervention, post-four weeks, and post-eight weeks. The data were analyzed using repeated measures ANOVA. The level of significance was considered at p-value<0.05.

Results

Participants with CMLBP significantly improved in all variables in all three groups (p-value≤0.05). On inter-group comparison, group A showed better improvement in lumbar extension range of motion than the other two groups, with a mean difference of MMST in group A of 0.62±0.30, group B of 0.52±0.22, and group C of 0.36±0.02, with a p-value ≤0.002. Group C showed more improvement in core strength, with a mean difference of 5.0±0.25 in group A, 3.2±0.56 in group B, and 5.2±0.57 in group C, with a p-value ≤0.03. A significant improvement was observed in NPRS, MMST flexion, RMDQ, and uncontrolled movements (UCMs).

Conclusion

All three methods of treatment are effective in the management of CMLBP. Clinically, kinetic control showed better improvement in reducing pain and improving lumbar flexion and extension range of motion. Functional disability was better improved with lumbar stabilization exercises, and core strength was improved with a combination of KC and LSE. However, a combination of MR and LSE helps improve core strength, and movement retraining improves lumbar extension.

## Introduction

Low back pain (LBP) is defined as discomfort and pain that is present between the costal margins and inferior gluteal fold. It may or may not be accompanied by pain referred to the leg. Back pain persisting for more than three months is referred to as chronic LBP (CLBP), as recommended by the National Institute of Health Task Force [1]. LBP has become a prime health concern, with a lifetime prevalence of about 70-85% globally. It has now become the major reason for disability and work absenteeism, which is leading to a huge pecuniary burden and indirectly causing deficits in productivity. Ferguson et al. reported that after iron deficiency anaemia in India, neck and back pain are major concerns for disability [2]. Patients with specific low back pain have symptoms that are caused by pathologies like infections, pathologies related to herniated discs, fractures, or osteoporosis that contribute to around 15% of all back pains, whereas LBP with symptoms with no clear specific cause is termed nonspecific or mechanical LBP (MLBP) [1].

An extensive review regarding the management of LBP has reported that exercise plays a prime role in treating patients with LBP [3]. However, the results regarding the appropriate type of exercise are the subject of controversy. Various subgroups clinically incorporate aspects of motor control in the assessment of LBP patients. van Dieën et al. developed and described the movement system impairment (MSI) classification system and explained that LBP patients prefer to move lumbar joints more easily when compared to other segments or adjoining joints (for example, thoracic joints or hips). This could lead to repeated movement patterns during daily living activities, thus, in due course, causing an exorbitant burden on tissues related to the specific joint [4]. Movement health is defined as a state that is not only injury-free and absent of the presence of uncontrolled movement (UCM) but also a state that allows the exerciser to choose how to move [5]. MSI is also termed ‘movement control impairment’ and ‘motor control dysfunction’ [6]. Subclassification of MSI involves the identification of mechanically based impairments and associated symptoms with a series of movement control (MC) tests that include lumbopelvic flexion, lumbopelvic extension, lumbopelvic open chain rotation, and lumbopelvic closed chain rotation [7]. When performing upper and lower limb movements, the MSI classification model assesses the patient's ability to maintain a stable lumbopelvic region [8]. The kinesiopathologic model (KPM) is the theoretical basis on which MSI syndromes work. As per this model, sustained alignments and repetitive movements can induce pathology. A joint that moves too easily many times is the site of pain generation, causing microinstability along with relative stiffness, thus movement occurring in the path of least resistance. Therefore, training focuses on correcting movements and alignments rather than targeting isolated muscles, which aids in inducing musculoskeletal and neural adaptations [9].

Lumbar stabilization exercises (LSEs) come under the broader category of ‘treatment-based classification’, and it was put forward by Delitto et al. [10]. Frequently used synonyms for lumbar stabilization exercises are ‘spine stabilization exercises’, ‘motor control exercises', and ‘core strengthening’. In CLBP, the stabilizing system, as explained by Punjabi, gets disturbed due to arthrogenic inhibition. There is inhibition of the neuromuscular control system by nociceptive signals, resulting in reduced neural drive to the muscles, thus affecting movement and stability [1]. Very few studies that have included subgroupings have reported positive outcomes [11,6]. Heat therapy in the form of hot packs and heat wraps has been reported to reduce muscle spasms, decrease stiffness, and reduce inflammatory changes, thereby increasing blood circulation to the area.

The difference in the movement control approach from LSE is that movement retraining (MR) focuses on the activation and integration of deep trunk muscles into complex dynamic, static, and functional tasks. It helps change movement behaviour with the combination of cognitive and physical learning processes instead of just strengthening a muscle group, whereas LSE is focused on the performance and function of core muscles, mainly transversus abdominis and multifidus [12]. There is an extensive dearth of literature as to which form of the above-mentioned exercise is superior to improving symptoms in mechanical LBP patients. The purpose of this study was to determine and compare the effects of MR, LSE, and combinations of both exercises on pain, flexibility, strength, and functional disability in chronic mechanical low back pain (CMLBP) patients.

## Materials and methods

Study design

It was a pilot study that was conducted in a tertiary care hospital in Pune, India. The research was approved by Dr. D. Y. Patil Vidyapeeth Institutional Ethics Committee with reference number DYPV/EC/450/2020 and registered with the Clinical Trial Registry of India (CTRI) with the registration number CTRI/2020/06/025813.

Participants

Fifteen patients with CMLBP were included in the study. The inclusion criteria were patients with low back pain for more than three months, age group 20-40 years, both genders, and those with mild and moderate pain on the Numerical Pain Rating Scale (NPRS). Exclusion criteria were history of spine surgery, spinal tumours, recent lower limb surgery, trauma to the back and lower extremities, radiating pain in the lower extremities, diagnosed pathological conditions related to low back pain, diagnosed cases of rheumatoid arthritis of the spine, ankylosing spondylitis, spinal deformities, neurological diseases, H/O cardiovascular disease, stroke, pregnancy, any gynaecological conditions leading to LBP, renal conditions, BMI >30 kg/m^2^, and participants taking any other forms of treatment for LBP. The objective of the study was well explained, and written informed consent in either English, Hindi, or Marathi was obtained from all the study participants.

Study procedure

**Figure 1:**
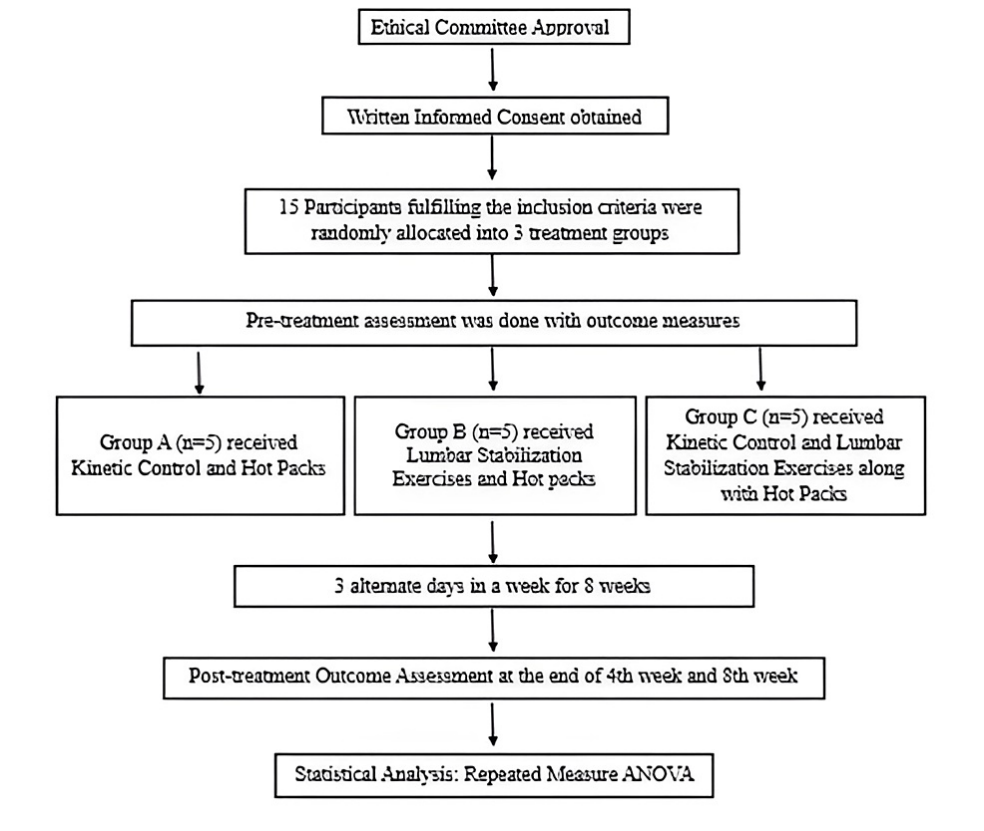
Flow chart showing the number of participants recruited during the study

Eligible participants fulfilling the inclusion criteria were allocated randomly into three groups using the lottery method. Group A (n=5) received MR and hot packs. Group B (n=5) received LSE and hot packs. Group C (n=5) received a combination of MR and LSE along with hot packs. All participants performed the exercise sessions under the supervision of a physiotherapist and were treated for three days a week for a period of eight weeks (Figure 1).

Intervention

Initially, a hot pack was placed over the lumbopelvic region in a prone position to help reduce spasms for 20 minutes prior to initiating exercises in each group.

Movement Retraining

**Figure 2:**
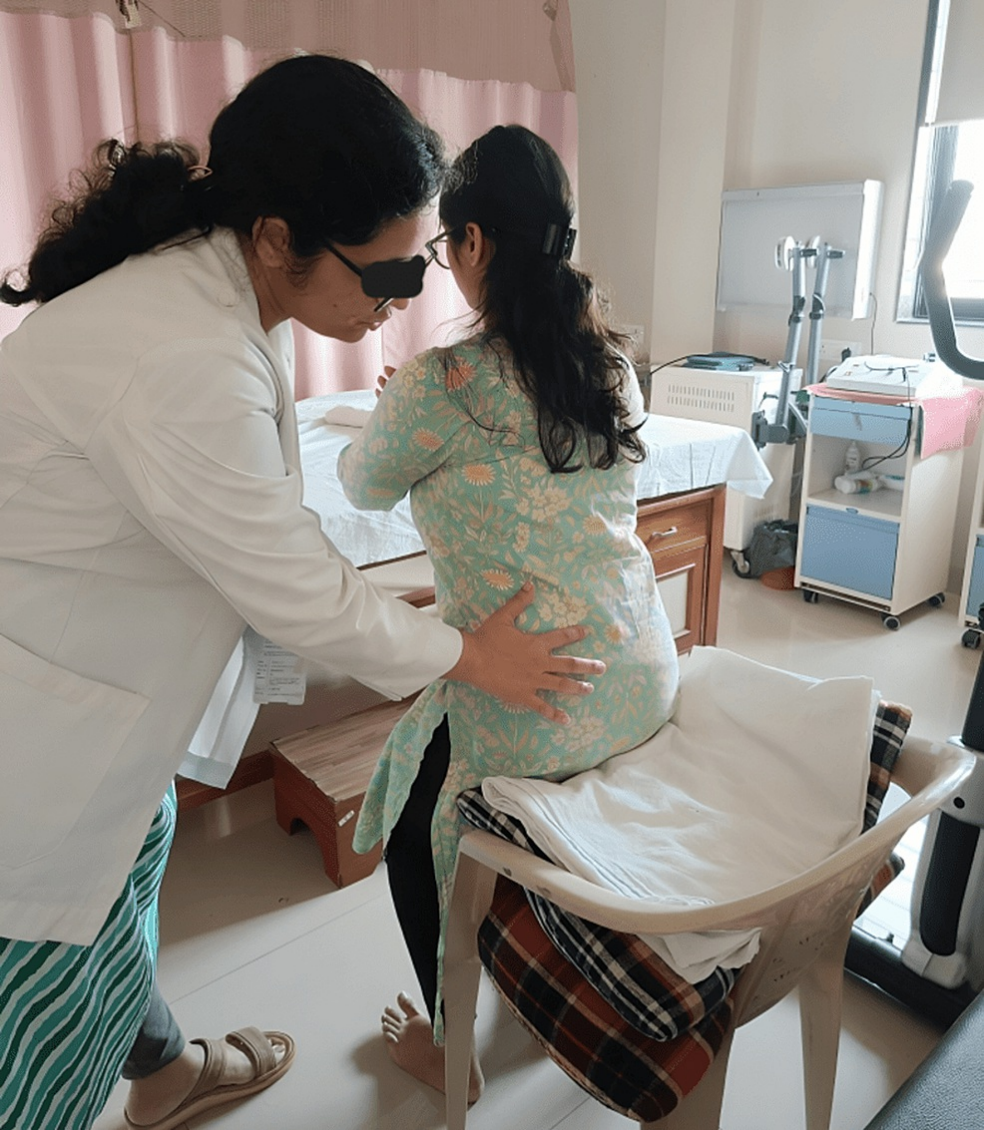
Movement retraining given to the patient

The direction and site of UCM were identified for each patient in terms of lumbopelvic flexion, lumbopelvic extension, lumbopelvic open chain rotation control, and lumbopelvic closed chain rotation control tests. A total of 28 tests were performed to assess UCM in groups A and C, with 7 tests performed for flexion UCM, 9 tests for extension UCM, and 12 tests for rotation UCM. The principles of procedure regarding cognitive movement control tests are explained in Table 1. The participant was considered eligible to pass the test if he/she had the potential to keep up the alignment at the desired site and direction while the region below or above was moved to achieve a preset benchmark [13]. MR was given as a tailored eight-week programme for all the tests that failed to achieve the preset benchmark. Exercises for identified UCM were given in a similar way as the testing but were performed for two minutes, and progression was given by challenging the positions cognitively (Table 1 and Figure 2) [7].

**Table 1:** Cognitive movement control tests: principles of procedure

Starting position	Neutral training region
Training the test movement	Train the test movement with different techniques
	Visual demonstration of the test
	Verbal explanation and description of the test movement
	Tactile feedback with adhesive tape
	Facilitation or guidance given to the participants through the test movement
Learning actively	Participant is informed to practice the movement with facilitation and feedback for 3 to 5 repetitions
Test	On understanding the test movement, participant is informed to perform the test to the benchmark with no feedback techniques
Rating	The therapist observes the test performance for uncontrolled movements at site and direction

Lumbar Stabilization Exercises

Participants in group B were treated with LSE [14] along with hot packs. Initially, all participants were treated with isometric holding exercises, abdominal hollowing in alternate static positions, and abdominal hollowing with challenges in different positions for four weeks. The progression of exercises from the fifth to eighth weeks was: pelvic floor contractions with bridging on a ball, single leg bridging with an unstable base of support, quadruped multifidus exercise, followed by planks that included half-side planks (knees flexed), side planks, and full-front planks.

**Figure 3:**
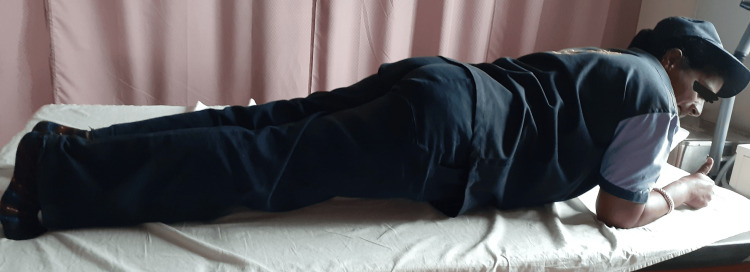
Participant performing lumbar stabilization exercises

Each exercise was performed for five repetitions with a 10-second hold, which was then progressed to 10 repetitions with a 10-second hold until the end of the fourth week. A new set of exercises was given from the fifth week onwards with the same number of repetitions. All participants were given five seconds of rest between each repetition and 30 seconds of rest between each exercise (Figure 3).

Participants in group C were treated with a combination of MR and LSE along with hot packs.

Outcome measures

Pain Intensity

Pain intensity was measured with an 11-point NPRS that is measured from 0 to 10. ‘0’ is no pain; 1-3 indicates mild pain; 4-6 indicates moderate pain; and 7-10 is the most severe pain imaginable. Here, the participant is assessed based on the marking made by him/her on the straight line [15]. It is found to be an easy, simple, and quick measure of pain intensity. It has also proven to have satisfactory reliability, sensitivity, and accuracy. Furthermore, it also quantifies the assessment of pain [16].

Range of Motion

**Figure 4:**
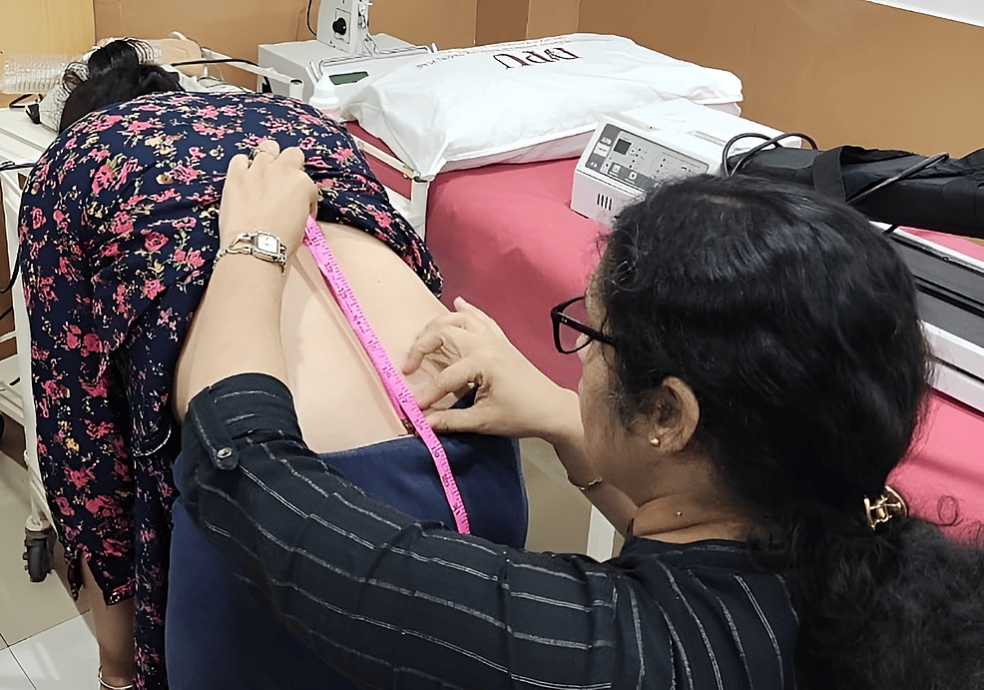
Lumbar flexion range of motion with Modified-Modified Schober's test

Lumbar flexion and extension were measured with the Modified-Modified Schober’s test (MMST). It has been reported as a gold standard for measuring lumbar range of motion (ROM). It shows excellent intra-rater (ICC = 0.95; 95% CI 0.89-0.97) and inter-rater (ICC = 0.91; 95% CI 0.83-0.96) reliability and moderate validity with r = 0.67; 95% CI 0.44-0.84. Initially, the inferior margins of the posterior superior iliac spine PSIS were palpated, and a mark was made horizontally with a marker from the midline of the lumbar spine to the PSIS. Another mark was made 15 cm above the marked point. The patient was then informed to actively bend forward from the trunk only until the pain in the lumbosacral region was appreciated. The new distance was measured between both points, following which the patient was informed to return to the starting position. The most relevant change between the starting distance in a neutral position and a flexed position was considered to be the amount of lumbar flexion [17], as shown in Figure 4. With the same landmarks, the procedure was repeated for lumbar extension ROM. The normative values for lumbar flexion ROM with MMST are 6.85±1.18 cm and 2.42±0.74 cm for lumbar extension ROM [18] (Figure 4).

Lumbar Core Strength

**Figure 5:**
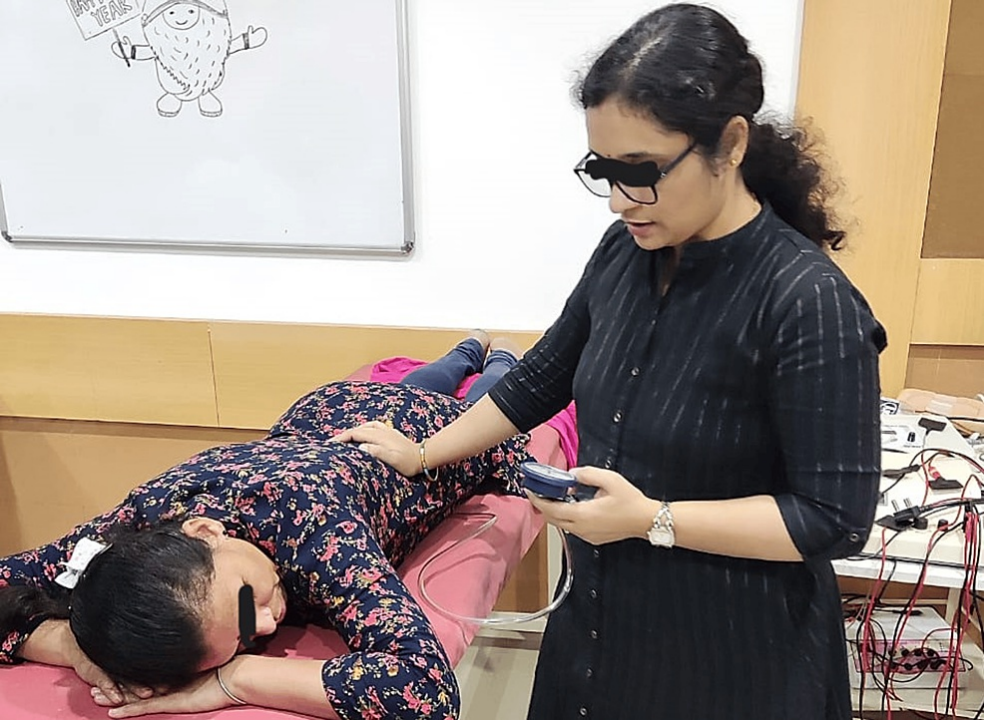
Measurement of core strength with pressure biofeedback unit

The strength of transversus abdominis muscle contraction was measured with a pressure biofeedback unit (PBU) (Stabilizer™, Chattanooga Group, Vista, CA, USA); the patient was taken in a prone position with the pad located under the abdomen, and the pressure biofeedback unit was inflated to 70 mmHg. The patient was then instructed to perform an abdominal drawing manoeuvre. The pressure change was noted, and the patient was informed to maintain the pressure for 10 seconds without any pelvic movement, as shown in Figure 5. The decrease in pressure from 4 to 10 mmHg is considered normal [19].

Roland Morris Disability Questionnaire

The Roland Morris Disability Questionnaire (RMDQ) is a valid and reliable tool for estimating the level of disability in LBP patients. It is composed of 24 questions that are to be answered by the patients with answers of either ‘Yes’ or ‘No’. The variation of the total score may range from a score of 0 (no disability) to a score of 24 (maximum disability). Validity and reliability have been tested, with reliability rated as high and internal and construct validity rated as good [20]. The questionnaire was administered in English, Hindi, and Marathi versions at baseline, post-four weeks, and post-eight weeks of intervention. The validity and reliability of all three language versions have been confirmed [21,22]. 

Movement Control Dissociation Tests

**Figure 6:**
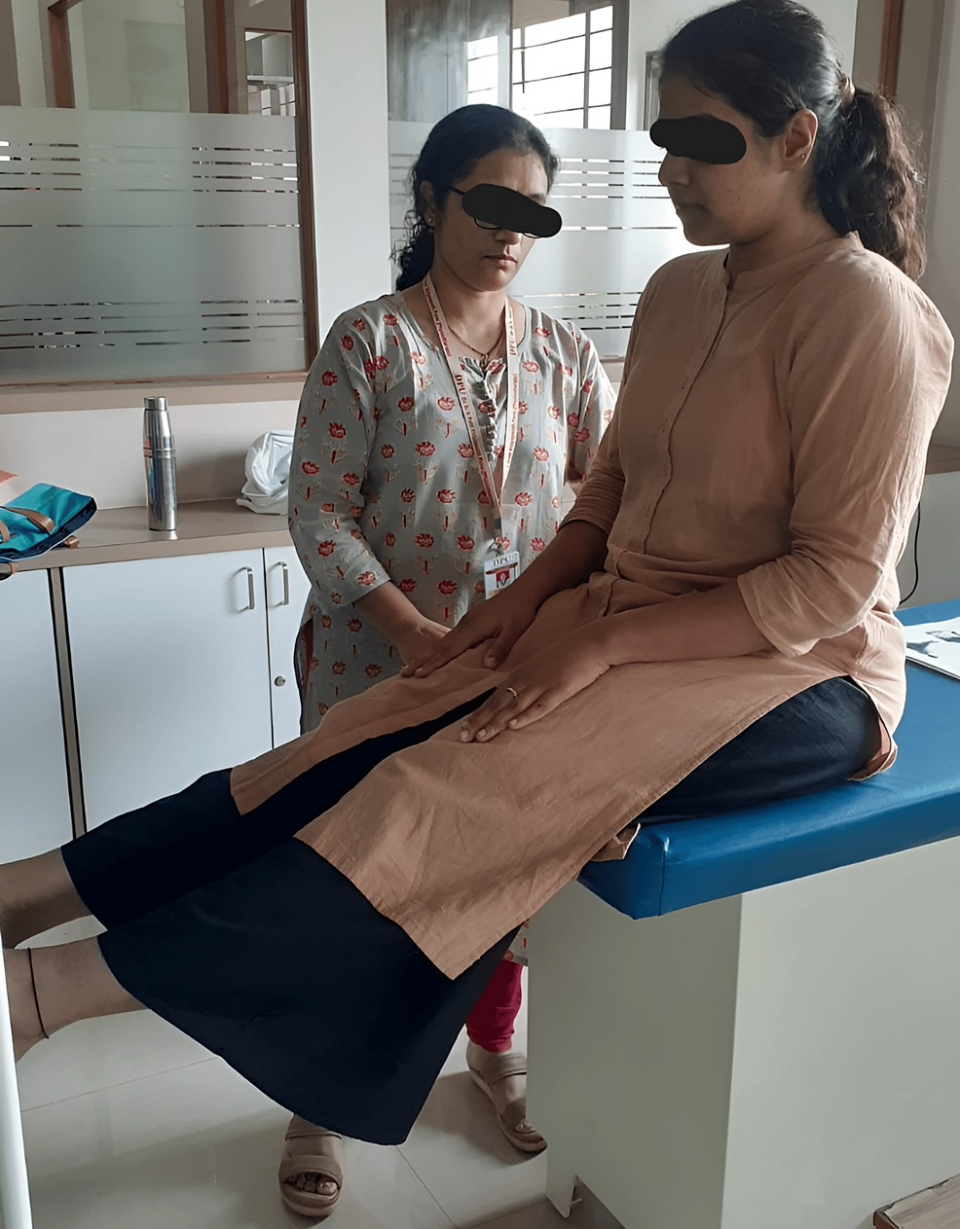
Assessment of patient for movement control dissociation test

Movement control impairments in all four directions were assessed for lumbopelvic flexion, lumbopelvic extension, lumbopelvic open chain rotation, and lumbopelvic close chain rotation. Identification of the direction and site of UCM was carried out with 28 cognitive motor control tests, and the tests that failed to clear were considered for retraining. The validity and reliability of tests that can identify dysfunction have been accepted. Also, the reliability of classifying the patients under the MSI model is reported to be moderate to excellent, with the kappa statistics ranging from 0.61 to 0.81 and a percentage ranging from 0.75% to 0.87% [23,24] (Figure 6).

All outcome measures were taken at baseline, post-four weeks, and post-eight weeks of intervention.

## Results

MedCalc software (version 18.2.1, Healthcare Technology, New York, USA) was used for data analysis and interpretation. Descriptive statistics like percentage, mean, and standard deviation were calculated for appropriate variables. After confirming the normal distribution of the data through the Shapiro-Wilk normality test, we conducted a further analysis using repeated measures ANOVA for within-group and between-group analyses. A nonparametric test of significance was used due to the non-normal distribution of the data for UCM. Within-group differences were compared using Friedman ANOVA, and between-group differences were compared with the Kruskal-Wallis H test. The p-value was set at 5%. All p-values less than 0.05 were treated as significant.

Table 2 shows the demographic data for all three groups (Tables 2-6). Fifteen participants (five per group) completed the treatment sessions for eight weeks, with three sessions per week. All three groups showed significant improvement (p-value ≤ 0.05) in all outcome measures when analyzed individually. This showed that all three interventions are effective in improving pain (Table 3), ROM (Table 4), core strength (Table 5), functional disability (Table 6), and MC. Also, MR and a combination of MR and LSE are effective in improving movement control. On inter-group comparison, significant improvement was observed in MMST extension (p-value=0.002) with a greater mean difference in group A and CS (p-value=0.03) with a greater mean difference observed in group C. However, no significant improvement was observed in NPRS, MMST flexion, RMDQ, and UCM. This shows that MR is more effective in improving lumbar extension ROM, and the combination of MR and LSE is more effective in improving CS as compared to the other two groups.

**Table 2:** Demographic details of group A, group B, and group C SD: standard deviation, BMI: body mass index

Gender	Group A	Group B	Group C	Percentage
Male	2	0	0	13%
Female	3	5	5	87%
Mean age (SD)	27.6±3.51	26.8±3.35	24±1.22	-
BMI	21.66±2.35	22.6±1.34	20.8±1.46	-

**Table 3:** Within-group and between-group analysis for pain in group A, group B, and group C *p < 0.05, SD: standard deviation, NPRS: Numerical Pain Rating Scale

Outcome measures	Group	Variable	Mean ± S.D.	Repeated measures	
				ANOVA (within-group)	ANOVA (between-group)
NPRS (at rest)	A	Pre	2.8 ± 2.28	F (2,24) = 26.89; P < 0.001*	F (2,12) = 1.27; P = 0.316
		Post 4 weeks	1.4 ± 0.83		
		Post 8 weeks	0.6 ± 0.89		
	B	Pre	1.6 ± 1.40	F (2,24) = 24.37; P < 0.001*	
		Post 4 weeks	0.4 ± 0.55		
		Post 8 weeks	0.2 ± 0.45		
	C	Pre	1.6 ± 0.55	F (2,24) = 33.36; P < 0.001*	
		Post 4 weeks	0.4 ± 0.54		
		Post 8 weeks	0.2 ± 0.45		
NPRS (on activity)	A	Pre	6.4 ± 1.14	F (2,24) = 151.75; P < 0.001*	F (2,12) = 0.95; P = 0.412
		Post 4 weeks	3.4 ± 1.14		
		Post 8 weeks	1.6 ± 0.55		
	B	Pre	5.2 ± 0.84	F (2,24) = 163.87; P < 0.001*	
		Post 4 weeks	3.2 ± 0.84		
		Post 8 weeks	1.4 ± 1.4		
	C	Pre	5.8 ± 0.84	F (2,24) = 142.36; P < 0.001*	
		Post 4 weeks	4.2 ± 0.84		
		Post 8 weeks	1.6 ± 0.90		

**Table 4:** Within-group and between-group analysis for MMST in group A, group B, and group C MMST: Modified Modified Schobers Test, SD: standard deviation, *p-value < 0.05

Outcome measures	Group	Variable	Mean ± S.D.	Repeated measures	
				ANOVA (within-group)	ANOVA (between-group)
MMST (flexion)	A	Pre	4.12 ± 0.69	F (2,24) = 117.01; P < 0.001*	F (2,12) = 1.16; P = 0.345
		Post 4 weeks	5.14 ± 0.49		
		Post 8 weeks	5.88 ± 0.65		
	B	Pre	4.78 ± 0.51	F (2,24) = 122.29; P < 0.001*	
		Post 4 weeks	5.36 ± 0.34		
		Post 8 weeks	6.2 ± 0.16		
	C	Pre	4.5 ± 0.41	F (2,24) = 139.73; P< 0.001*	
		Post 4 weeks	5.16 ± 0.44		
		Post 8 weeks	6.1 ± 0.37		
MMST (extension)	A	Pre	2.12 ± 0.16	F (2,24) = 27.77; P < 0.001*	F (2,12) = 10.60; P = 0.002*
		Post 4 weeks	2.12 ± 0.18		
		Post 8 weeks	2.74 ± 0.46		
	B	Pre	1.94 ± 0.89	F (2,24) = 43.13; P < 0.001*	
		Post 4 weeks	2.06 ± 0.89		
		Post 8 weeks	2.46 ± 0.11		
	C	Pre	1.94 ± 0.09	F (2,24) = 31.72; P < 0.001*	
		Post 4 weeks	2.04 ± 0.05		
		Post 8 weeks	2.3 ± 0.07		

**Table 5:** Within-group and between-group analysis for core strength in group A, group B, and group C *p-value < 0.05, SD: standard deviation

Group	Variable	Mean ± S.D.	Repeated measures	
			ANOVA (within-group)	ANOVA (between-group)
A	Pre	3.4 ± 0.89	F (2,24) = 178.79; P < 0.001*	F (2,12) = 4.32; P = 0.03*
	Post 4 weeks	5.4 ± 0.55		
	Post 8 weeks	8.4 ± 1.14		
B	Pre	3.4 ± 0.58	F (2,24) = 123.51; P < 0.001*	
	Post 4 weeks	4.6 ± 0.54		
	Post 8 weeks	6.6 ± 1.14		
C	Pre	3.8 ± 0.84	F (2,24) = 129.24; P < 0.001*	
	Post 4 weeks	6.2 ± 1.09		
	Post 8 weeks	9 ± 1.41		

**Table 6:** Within group and between group analysis for RMDQ *p-value < 0.05, SD: standard deviation, RMDQ: Roland Morris Disability Questionnaire

Group	Variable	Mean ± SD	Repeated measures	
			ANOVA (within-group)	ANOVA (between-group)
A	Pre	6.6 ± 1.52	F (2,24) = 126; P < 0.001*	F (2,12) =0.42; P = 0.66
	Post 4 weeks	4.4 ± 1.51		
	Post 8 weeks	2.2 ± 0.84		
B	Pre	6.6 ± 1.34	F (2,12) = 102.90; P < 0.001*	
	Post 4 weeks	4.4 ± 1.14		
	Post 8 weeks	1.4 ± 0.54		
C	Pre	5.8 ± 1.30	F (2,12) = 94.36; P < 0.001*	
	Post 4 weeks	4.2 ± 0.84		
	Post 8 weeks	1.6 ± 0.55		

## Discussion

The present pilot experimental study was conducted to compare the effects of movement retraining, lumbar stabilization exercises, and a combination of movement retraining and lumbar stabilization exercises on pain, flexibility, core strength, functional disability, and movement impairment in participants with chronic mechanical low back pain over a period of eight weeks. The outcome measures used were NPRS, MMST for lumbar flexion, and MMST for lumbar extension, PBU, RMDQ, and movement control tests.

The present study reported significant improvement in pain in all three groups when a hot pack was administered. On applying hot packs, there is activation of sensitive nerve endings, thus blocking the pain signals in the dorso-lumbar fascia and spinal cord. Also, there is an increase in tissue temperature, causing vasodilation and increased metabolism. Thus, the healing process is accelerated by removing pain-inducing mediators and enhancing oxygen in the tissues. The reason for a significant decrease in pain and improvement in functional disability in group A could be due to retraining of the abnormal movement patterns as identified by MSI, which could be the reason to reduce stress on the tissues that contribute to LBP [7]. The results of the present study are similar to those of Ahmed et al., wherein MR, along with a physical training programme, was given for a period of eight weeks. The study showed significant improvement in pain intensity, disability, and trunk flexion ROM in chronic LBP patients with radiculopathy [25]. Similarly, several research studies that included movement control reported improvement in terms of pain as well as activity level. Various feedback techniques were used to teach and facilitate the retraining movement, involving kinaesthetic feedback with adhesive tape, verbal instruction, and correction with pressure biofeedback, which was used as monitoring equipment. Also, effective cueing might have led to improved recruitment of TA and MF, thus causing pain relief and a decrease in disability [7].

Table 6 shows a decrease in pain and an improvement in disability after eight weeks of intervention with LSE. These exercises decrease the load on the passive subsystem, which includes the lumbar vertebrae and its ligaments and joint capsules, as explained by Akhtar et al. Pain reduction could also be due to improvements in lumbar stabilizer muscle function, thereby contributing to trunk postural control [26]. Also, Wattananon et al. explained that the active subsystem is the requirement for static and dynamic stability of the lumbar spine, and that stability is affected if core muscle contraction is weak, thus leading to LBP [27]. Further with LSE, there is coordinated activity of transversus abdominis, pelvic floor muscles, and diaphragm, creating a space to act like a balloon, thus dissociating diaphragm and pelvic floor muscles. Thus, there is a reduction in pain as the compressive load on the spine decreases, creating distraction of the lumbar spine.

A combination of MR and LSE also showed significant improvement in terms of pain and disability after eight weeks of intervention, with a p-value ≤ 0.001. A combination of both modes of exercise along with hot packs could be the reason for improved core muscle strengthening and lumbar spine ROM, thus augmenting pain relief and improving functional ability.

When an inter-group comparison was made, there was no significant difference in improvement observed in all three groups in terms of pain and disability. Both approaches, MR as well as LSE, work on the stabilization effect of the spine and involve combined and isolated limb and trunk movements, thus improving trunk control in patients. The results of the present study are in agreement with Henry et al., who compared movement control retraining with LSE for six weeks and a follow-up after 12 months post-treatment [28]. Also, Jacobs et al. mentioned that no exceptional results were obtained with MR in terms of pain and disability when compared with strengthening exercises. Hence, it was concluded that pain reduction and a decrease in disability could be the result of the overall effect of general exercises [4]. Since MR and LSE work on trunk control and improving spinal stability, this could be one of the reasons for pain relief, thus improving spinal ROM. Also, the application of hot packs causes transient receptor potential Vanilloid 1 (TRPV1), a receptor in the brain that regulates anti-nociceptive pathways, to get activated. This helps reduce muscle spasms and pain and thereby improve flexibility. Significant improvement in core muscle strength was observed in all three groups after eight weeks of intervention. Due to low load MR, there is activation of low threshold motor units, which could be the reason for the improvement in strength. Gwak et al. reported that there is a positive correlation between the cross-sectional area (CSA) of core muscles and muscle strength and endurance. They also concluded that core muscle training leads to improved core strength and, hence, improves CSA [29]. With strength training, there is an increase in actin, myosin, and collagen in a muscle with a good amount of contractile and connective tissues that contribute to a larger CSA, causing an increase in muscle stiffness. Thus, it works as a primary control mechanism to stabilize the spine. Hence, it proves that a larger CSA of core muscles could be the reason for increased spinal stability. Also, Danneels et al. mentioned that the incorporation of only low-load training exercises is not sufficient to restore muscle CSA. Progressive overload training needs to be given to improve motor patterns and reduce pain and disability [30]. The findings in the literature well confirm the result of the present study that strength training should be included in the intervention of chronic LBP patients in addition to low-load activation patterns. On intergroup comparison, the present study showed significant improvement with the combination of the MR and LSE groups, with a mean difference of 5.2 mm Hg, indicating better improvement of TA strength due to the corset effect as compared to individualized treatment groups. Thus, the combination of both treatments might have led to improved core muscle strength. Experimental studies have proved that a pressure reduction of 4 mmHg in TA activity suggests good strength of contraction [19].

The limitations of this study were that the male and female ratios in the study were not equal in all three groups. Also, surface electromyography (sEMG) could have been used to find the effects of exercises on core muscle activation in chronic mechanical low back pain patients. This study contributes to sample size calculations for performing an experimental study on a large sample size. Also, further studies can be done to analyze and treat movement impairment in the lumbopelvic region in people associated with different occupations involving static or repetitive movements as a precautionary measure to prevent low back pain in the future.

## Conclusions

The results of this pilot study show that movement retraining, lumbar stabilization exercises, and a combination of both exercise groups have beneficial effects in terms of pain, disability, lumbar range of motion, core strength, and movement control impairments among chronic mechanical low back pain patients. However, a combination of both of these exercises has better effects on improving core strength, as movement retraining helps in activation and lumbar stabilization exercises help in strengthening core muscles. Also, movement retraining is more beneficial for improving the lumbar extension range of motion.
